# MRI-identified abnormalities and wrist range of motion in asymptomatic versus symptomatic computer users

**DOI:** 10.1186/1471-2474-11-273

**Published:** 2010-11-25

**Authors:** Ronald A Burgess, William F Pavlosky, R Terry Thompson

**Affiliations:** 1 Department of Medical Biophysics, University of Western Ontario, London, Ontario, Canada; 2 Imaging Division, Lawson Health Research Institute, London, Ontario, Canada; 3 Diagnostic Imaging, St. Joseph's Health Care, London, Ontario, Canada

## Abstract

**Background:**

Previous work has shown an association between restricted wrist range of motion (ROM) and upper extremity musculoskeletal disorders in computer users. We compared the prevalence of MRI-identified wrist abnormalities and wrist ROM between asymptomatic and symptomatic computer users.

**Methods:**

MR images at 1.5 T of both wrists were obtained from 10 asymptomatic controls (8 F, 2 M) and 14 computer users (10 F, 4 M) with chronic wrist pain (10 bilateral; 4 right-side). Maximum wrist range of motion in flexion and radioulnar deviation was measured with an electrogoniometer.

**Results:**

Extraosseous ganglia were identified in 66.6% of asymptomatic wrists and in 75% of symptomatic wrists. Intraosseous ganglia were identified in 45.8% of asymptomatic wrists and in 75% of symptomatic wrists, and were significantly (p < .05) larger in the symptomatic wrists. Distal ECU tendon instability was identified in 58.4% of both asymptomatic and symptomatic wrists. Dominant wrist flexion was significantly greater in the asymptomatic group (68.8 ± 6.7 deg.) compared to the symptomatic group (60.7 ± 7.3 deg.), *p *< .01. There was no significant correlation between wrist flexion and intraosseous ganglion burden (p = .09)

**Conclusions:**

This appears to be the first MRI study of wrist abnormalities in computer users.

This study demonstrates that a variety of wrist abnormalities are common in computer users and that only intraosseous ganglia prevalence and size differed between asymptomatic and symptomatic wrists. Flexion was restricted in the dominant wrist of the symptomatic group, but the correlation between wrist flexion and intraosseous ganglion burden did not reach significance. Flexion restriction may be an indicator of increased joint loading, and identifying the cause may help to guide preventive and therapeutic interventions.

## Background

Computer use is often associated with an increased prevalence of hand and wrist disorders [1-6], but the relationship remains controversial due to the frequent absence of identifiable pathology [7-12]. Symptoms are thought due to soft-tissue injury resulting from prolonged repetitive hand use, and have been referred to as repetitive strain injury, cumulative trauma disorder, occupational overuse syndrome, or work-related upper extremity musculoskeletal disorder. Symptoms may be associated with specific clinical entities such as peripheral nerve entrapment, extraosseous ganglia, tendon or muscle disorders, or may be non-specific [13-15]. Magnetic resonance imaging (MRI) has been used to study heterogeneous groups of patients with specific disorders, such as carpal tunnel syndrome [16-19], and lateral epicondylitis [20], as well as unexplained wrist pain [21,22]. To the best of our knowledge there are no previously published MRI studies of computer users with chronic wrist pain.

A number of risk factors other than repetitive hand use have been implicated including gender, psychological stress, and other psychosocial factors [23-25]. Previous studies have found that symptomatic computer users exhibit limited wrist range of motion attributed to increased antagonist wrist motor muscle tension [4,23,26]. Increased wrist motor muscle tension increases biomechanical stress within the wrist joint, and this additional stress may become clinically symptomatic [4].

The aims of this study were twofold:

1) Compare the prevalence of MRI-identified abnormalities in the wrists of symptomatic versus asymptomatic computer users. The hypothesis is that the prevalence of abnormalities will be greater in the symptomatic wrists compared to the asymptomatic wrists.

2) Compare wrist range of motion between symptomatic and asymptomatic computer users. The hypothesis is that wrist flexion will be decreased in the symptomatic group.

## Methods

The study was approved by the local ethics review committee, and all subjects provided informed consent. Advertisements were posted at a local university, hospital, occupational health and safety office, and on our institute's website recruiting subjects who used a computer for a minimum of 4 hours per day for at least 3 years, and had either no history of wrist pain, or wrist pain for at least 6 months duration.

Inclusion criteria: 1) Daily computer use for a minimum of 4 hours per day for at least 3 years. 2) No history of upper limb symptoms to define asymptomatic. 3) Wrist pain for at least 6 months to define symptomatic.

Fourteen symptomatic computer users (10 female, 4 male), mean age = 38.4 (±8.4 yrs., range = 25 - 49 yrs.), and 10 asymptomatic computer users (8 female, 2 male) mean age = 39.4 (±8.7 yrs., range = 28 - 50 yrs.) were recruited for the study. The subjects answered a questionnaire describing their age, gender, occupation, dominant hand, number of years and hours per day of computer use, work absence due to symptoms, and any history of upper limb trauma. Symptomatic subjects were asked to identify the location of their upper limb symptoms on a hand/forearm diagram. The subject recorded the symptom characteristics (pain, numbness, tingling, or decreased sensation), intensity (mild, moderate, or severe), frequency (sometimes, often, or always), and the number of months since symptom onset.

MR images of both wrists were acquired with a Siemens Espree 1.5 T scanner using an 8-channel wrist coil. The subjects were imaged in the "superman" position with the arm over the head while lying prone. The wrist was in neutral radioulnar and flexion/extension posture, the elbow posture was in extension, and the forearm posture varied between neutral and supine.

The following fast spin echo and gradient echo (GRE) imaging sequences were used:

Axial T1 (TR 495/TE 16 ms); 320 × 320 matrix; 3 mm thick; 0.3 mm gap;

Coronal T1 (TR 545/TE 16 ms); 320 × 320 matrix; 2 mm thick; 0.2 mm gap;

Coronal proton density with fatsat (TR 3700 TE 33 ms); 320 × 320 matrix; 2 mm thick; 0.2 mm gap;

Axial Inversion Recovery (TR/TE/TI = 5740/39/140 ms); 256 × 256 matrix; 3 mm thick; 0.3 mm gap;

Coronal Inversion Recovery (TR/TE/TI = 6310/39/140 ms); 256 × 256 matrix; 2 mm thick; 0.2 mm gap;

Sagittal Inversion Recovery (TR/TE/TI = 5480/35/140 ms); 272 × 320 matrix; 3 mm thick; 0.3 mm gap;

(GRE) Coronal T2 (TR 564/TE 24 ms); 230 × 256 matrix; 2 mm thick; 0.2 mm gap;

A field of view of 90 mm × 90 mm was used for all sequences. The MR images were assessed by a radiologist (W.P.) with 25 year's experience who was blinded to the subjects' symptom status. The following criteria were used to identify the presence of ganglia. A well-marginated region of fluid-like signal (i.e. hyperintense with a STIR-w sequence and hypointense with a T1-w sequence) was diagnosed as an intraosseous ganglion when located within bone, or as an extraosseous ganglion when located in soft tissue. The maximum area of intraosseous and extraosseous ganglia was measured using Onis Dicom Viewer, ver. 2 (DigitalCore, Tokyo, Japan) by manually delineating the outer boundary of the ganglia in the imaging plane showing the greatest area.

Range of motion data were acquired using a Biometrics (Gwent, UK) SG65 dual axis goniometer and ADU301 angle display unit. The goniometer data was digitized with a Dataq Instruments (Akron, OH, USA) DI-158U analog to digital converter. Measures of maximum active wrist range of motion during flexion and radioulnar deviation were acquired with the forearm prone and elbow fully extended. Maximum wrist range of motion was taken as the maximum of three consecutive measures. Previous work has shown that wrist flexion is affected by forearm posture [26,27]. Single measures of maximum active wrist flexion with the forearm prone and supine were acquired with the forearm vertical (i.e., elbow flexed approximately 90 degrees). This forearm position allowed accurate repositioning and zeroing of the goniometer, which was required to prevent crosstalk with forearm supination.

The distal end of the goniometer was affixed to the dorsum of the hand aligned with the third metacarpal. The proximal end was affixed to the dorsum of the forearm aligned with the radius for measurements with the forearm prone. For measurements with the forearm supine, the proximal end was affixed along a line extending from the 3^rd ^metacarpal to the midline of the olecranon. The display unit was zeroed with the forearm in neutral flexion/extension and radioulnar deviation. The subjects were instructed to flex their wrist as far as possible while keeping their wrist in neutral radioulnar deviation by maintaining the degree of radioulnar deviation on the angle display unit as close to zero as possible. The subjects were then instructed to radioulnar deviate their wrist as far as possible while maintaining the degree of flexion/extension on the angle display unit as close to zero as possible.

Statistical analysis was performed using SPSS ver. 17 (SPSS Inc., Chicago, IL, USA) with an alpha level of .05 for all tests. Computer exposure and wrist range of motion data were analyzed with independent t-tests. Comparisons of ganglia prevalence between asymptomatic and symptomatic wrists were performed with the chi-square test. Ganglia size was not normally distributed, and was compared with the Mann-Whitney U test. Correlation between wrist flexion and intraosseous ganglion burden was tested with Spearman's rank correlation coefficient.

## Results

### Questionnaire Results

The majority of the subjects' occupations involved clerical work (n = 14) or computer programming (n = 5), and all subjects were right-dominant. There was no significant difference in the number of years of computer exposure between the control group (13.1 ± 6.5 yrs.) and the symptomatic group (10.3 ± 5.1 yrs.), *p *= .35. The symptomatic subjects reported chronic wrist pain with a mean duration of 48 ± 36 months that was bilateral in 10 subjects and right-side only in 4 subjects, for a total of 24 symptomatic wrists. Only 1 subject reported work absence (6 years) due to symptoms. Previous adolescent wrist fracture/sprain was reported by 2 asymptomatic and 3 symptomatic subjects. Two symptomatic subjects had previous right wrist surgery: extensor carpi radialis brevis and flexor carpi radialis tendon release in one, and flexor retinaculum release in the other.

### Symptom Reports

Pain symptoms ranged between mild and severe, and were more pronounced in the dominant right wrist. Dorsal pain sites were reported in 15 wrists (8 radial, 5 central, 4 diffuse). Volar pain sites were reported in 11 wrists (6 diffuse, 2 central, 3 ulnar). Paresthesia in one or more of the digits 2-5 was reported in 11 wrists, and thenar paresthesia was reported in 5 wrists. Muscle pain (myalgia) was reported in the wrist extensors in 10 wrists and in the wrist flexors in 2 wrists.

### Extraosseous Ganglia

Extraosseous ganglia were identified in 16 asymptomatic and 18 symptomatic wrists (Table 1). There was no significant difference in the median area of extraosseous ganglia between the asymptomatic and symptomatic wrists. The most frequent location was adjacent to the pisotriquetral joint in the asymptomatic wrists (26%), and volar to the radioscaphoid articulation in symptomatic wrists (39.1%).

**Table 1:** Extraosseous ganglia (EOG) distribution and size.

LOCATION	ASYMPTOMATIC	SYMPTOMATIC
Dorsal	5	10
Volar	15	12
Distal radioulnar joint	3	1

# Wrists with EOG	6 right, 10 left	9 right, 9 left
# EOG in right wrist	9	16
# EOG in left wrist	14	7
Mean max. dimension (mm)	7.3 ± 4.0	8.4 ± 3.6
Mean max. area (mm^2^)	27.0 ± 17.9	32.9 ± 30
Median max. area (mm^2^)	23.3	23.6

### Intraosseous Ganglia

Intraosseous lesions exhibiting a fluid-like MR signal and located adjacent to ligament attachment sites (entheses) were identified in 11 asymptomatic wrists and 18 symptomatic wrists (Table 2). The imaging characteristics of these lesions are consistent with intraosseous ganglia (IOG). The prevalence of intraosseous ganglia in the symptomatic wrists (75%) was significantly greater than in the asymptomatic wrists (45.8%), χ^2 ^(1, n = 48) = 4.27, *p *= .039. The median maximum area of the intraosseous ganglia in the symptomatic wrists (Mdn = 7.5 mm^2^) was significantly greater than the asymptomatic wrists (Mdn = 5.0 mm^2^), U(1) = 526, Z = 2.02, *p *= .043.

**Table 2:** Intraosseous ganglia distribution and size.

BONE	# IOG Asymptomatic	# IOG Symptomatic
lunate	7	13
capitate	8	6
scaphoid	2	4
trapezoid	3	2
triquetrum	2	2
trapezium	1	2
hamate	2	2
distal radius	0	1
1st metacarpal base	1	0

Right wrist	11	14
Left wrist	14	18

Total	25	32

Mean max. dim. (mm)	1.9 ± 1.1	3.0 ± 3.2

Mean max. area (mm^2^)	6.1 ± 3.2	11.2 ± 11.1
Median max. area (mm^2^)	5	7.5*
Number of wrists	4 Right, 7 Left	9 Right, 9 Left

*p < .05

The distribution of intraosseous ganglia was similar between groups, and the majority were located in the lunate and capitate in both the asymptomatic (60%) and symptomatic (57.5%) wrists. One asymptomatic subject had 14 intraosseous ganglia (8 right), and one symptomatic subject had 7 intraosseous ganglia (5 left). Intraosseous and extraosseous ganglia originating at the same site were identified in 1 asymptomatic and 4 symptomatic wrists.

The carpal ligaments located immediately adjacent to the intraosseous ganglia are listed in Table 3. The most frequent site was adjacent to the extrinsic ligament attachment site of the dorsal lunate. Three intraosseous ganglia in the triquetrum were located adjacent to the attachment of the sheath of the distal extensor carpi ulnaris (ECU) tendon.

**Table 3:** Intraosseous ganglia distribution by ligament.

	ASYMPTOMATIC			SYMPTOMATIC		
	**Intrinsic**	**Extrinsic**		**Intrinsic**	**Extrinsic**	
	**Ligament**	**Ligaments**		**Ligament**	**Ligaments**	
**BONE**		**Dorsal**	**Volar**		**Dorsal**	**Volar**

lunate	S-L(3)	3	1	LT(3)	10	

capitate	C-Tra(2); C-H(5)			C-Tra(2);S-C(2);C-H(2)		

scaphoid	C-S(2)			C-S(4)		

trapezoid	C-Tra(2)			C-Tra(3)		

triquetrum		ECU(2)			ECU(1)	

trapezium	CMC(1)			CMC(2)		

hamate	C-H(3)			C-H(2)		

distal radius					LRL(1)	

1st metacarpal	CMC(1)					

Note. Name (number) of the ligaments located adjacent to the intraosseous ganglia in the asymptomatic and symptomatic wrists. (C-H, capitohamate ligament; C-S, capitoscaphoid ligament; C-Tra, capitotrapezoid ligament; CMC, carpometacarpal ligament; ECU, attachment of ECU tendon sheath on the triquetrum; LRL, long radiolunate ligament; L-T, lunatotriquetral ligament; S-L, scapholunate ligament).

**Figure 1:**
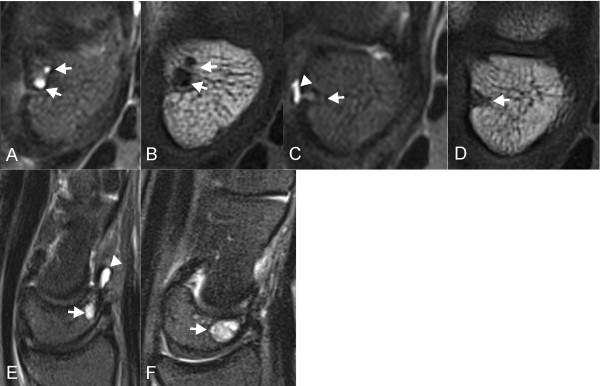
**Intraosseous ganglia of the dorsal lunate**. Axial STIR-w images (A, C) and axial T1-w images (B, D) show intraosseous ganglia (arrows) in the dorsal lunate at the attachment of the dorsal extrinsic ligaments. Sagittal STIR-w images (E, F) of intraosseous ganglia in the dorsal lunate. Images (A, B, E) are of symptomatic wrists, and images (C, D, F) are of asymptomatic wrists. Dorsal extraosseous ganglia (arrowheads) originating at the same site are shown in C and E.

**Figure 2:**
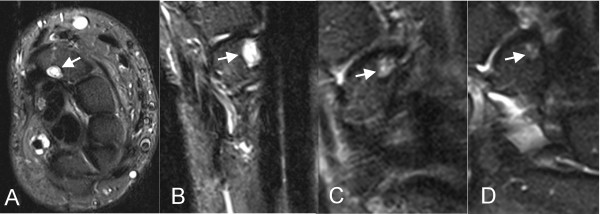
**Intraosseous ganglia of the distal scaphoid**. (A) Axial and (B) coronal STIR-w images showing an intraosseous ganglion (arrow) in the distal scaphoid adjacent to the capitoscaphoid ligament in a symptomatic wrist. Similar distal scaphoid ganglia are shown in coronal STIR-w images of a (C) symptomatic and (D) asymptomatic wrist.

**Figure 3:**
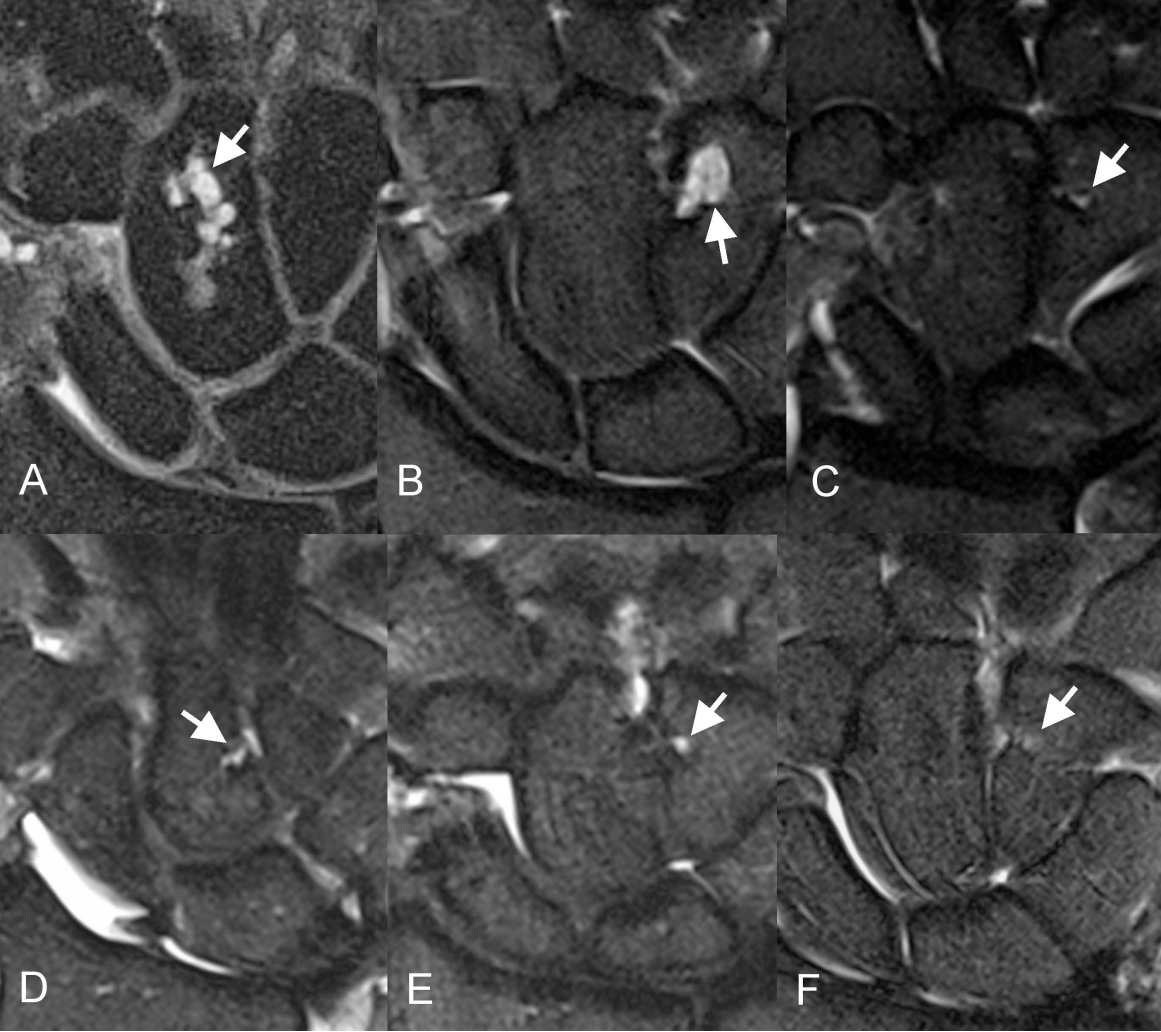
**Intraosseous ganglia of the capitate and hamate**. Coronal STIR-w images showing intraosseous ganglia (arrows) in the (A) capitate, and (B, C) hamate of symptomatic wrists; (D) capitate and (E, F) hamate of asymptomatic wrists

Examples of intraosseous ganglia in the dorsal lunate adjacent to the attachment of the dorsal extrinsic ligament in symptomatic and asymptomatic wrists are shown in Figure 1. The capitolunate angle of the asymptomatic subject (Figure 1-F) was not abnormal (>30 degrees). Figure 2 shows examples of intraosseous ganglia in the distal scaphoid adjacent to the attachment of the capitoscaphoid ligament of both symptomatic and asymptomatic wrists. Examples of intraosseous ganglia in the capitate and hamate for both symptomatic and asymptomatic wrists are shown in Figure 3. The subject with an intraosseous ganglion of the capitate shown in Figure 3-A had previous surgical release of the flexor carpi radialis and extensor carpi radialis brevis tendons.

### Tendinopathies

**Figure 4:**
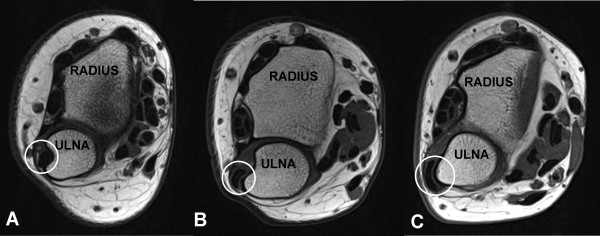
**Subluxation of the distal extensor carpi ulnaris (ECU) tendon**. Axial T1-w images with the forearm neutral showing the (A) normal position of distal ECU tendon (circle); (B) subluxed; (C) dislocated.

Mild tenosynovitis of the wrist extensors was identified in 2 asymptomatic and 5 symptomatic wrists, and most often affected the extensor carpi radialis brevis and longus tendons. Subluxation of the distal ECU tendon, as shown in Figure 4-B, was evident in 14 asymptomatic wrists and 14 symptomatic wrists. In 8 of these wrists (7 asymptomatic) the distal ECU tendon was completely dislocated volar to the ulnar border of the ulnar sulcus as shown in Figure 4-C.

### Persistent Median Artery

**Figure 5:**
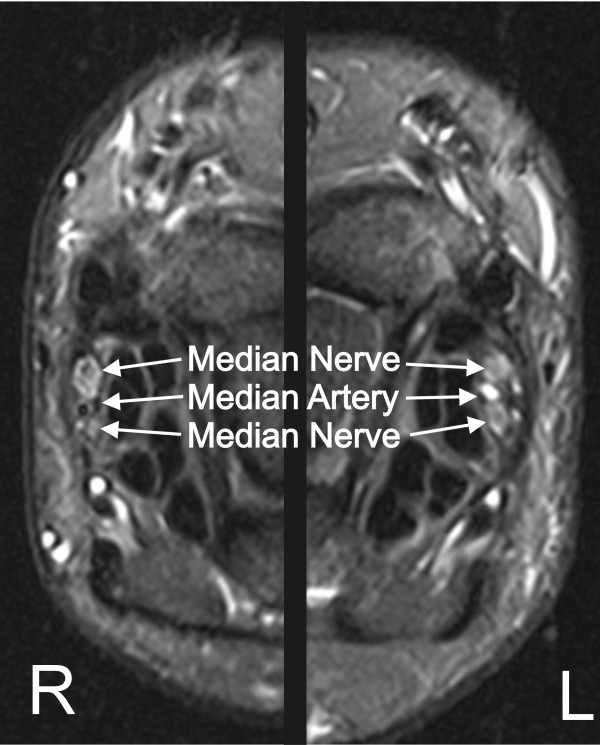
**MR image of bilateral persistent median artery**. Axial STIR-w images of the right and left wrists of a subject with bilateral CTS symptoms showing a persistent median artery within the bifurcated median nerve.

A persistent median artery was identified bilaterally in 1 asymptomatic subject and in 1 subject with symptoms consistent with carpal tunnel syndrome (CTS). The artery was located adjacent to the median nerve in the asymptomatic subject, and within a bifurcation of the median nerve in the symptomatic subject as shown in Figure 5.

### Other MRI Findings

Other MRI findings included mild-moderate radiocarpal joint effusion (2 asymptomatic, 1 symptomatic wrist), perforation or central thinning of the triangular fibrocartilage (4 asymptomatic wrists), areas of edema and/or sclerosis (1 asymptomatic, 2 symptomatic wrists), low signal lesion in the volar lunate suggestive of granulation within a ganglion (1 symptomatic wrist), partial scapholunate ligament tear (1 symptomatic wrist), and humpback scaphoid (1 asymptomatic wrist). There was no evidence of degenerative joint disease in any wrist.

### Wrist Range of Motion

The wrist range of motion data for the right and left wrists is shown in Table 4. Mean right wrist flexion in the asymptomatic group (68.8 ± 6.7 deg.) was significantly greater than the symptomatic group (60.7 ± 7.3 deg.), *p *= .007. During testing one symptomatic subject reported pain at the limit of wrist flexion.

**Table 4:** Wrist range of motion.

		Asymptomatic	Symptomatic
	**Motion/Forearm Posture**	**(n = 10)**	**(n = 14)**

		**(deg. ± SD)**	**(deg. ± SD)**

Right	Flexion/Prone	68.8 ± 6.7	60.7 ± 7.3**
	Ulnar Deviation/Prone	11.7 ± 6.4	8.9 ± 4.0
	
	ΔFlexion/Prone-Supine	3.5 ± 5.4	8.0 ± 11.9
	Ulnar Deviation	11.9 ± 7.9	10.1 ± 7.1
	
	Radioulnar Deviation/Prone	61.7 ± 9.3	60.1 ± 10.8

Left	Flexion/Prone	69.6 ± 9.2	61.7 ± 11.6
	Ulnar Deviation/Prone	11.7 ± 6.3	8.4 ± 6.1
	
	ΔFlexion/Prone-Supine	2.6 ± 8.4	8.3 ± 10.8
	Ulnar Deviation/Supine	10.6 ± 9.3	12.1 ± 9.3
	
	Radioulnar Deviation/Prone	62.2 ± 11.3	61.1 ± 8.8

Note. The first entry lists the mean maximum active flexion measured with the forearm prone followed by the degree of ulnar deviation at the flexion limit. Listed next is the mean difference in flexion between the prone and supine forearm postures followed by the degree of ulnar deviation at the flexion limit with the forearm supine. The final entry lists the degree of radioulnar deviation. ** *p *< .01.

**Figure 6:**
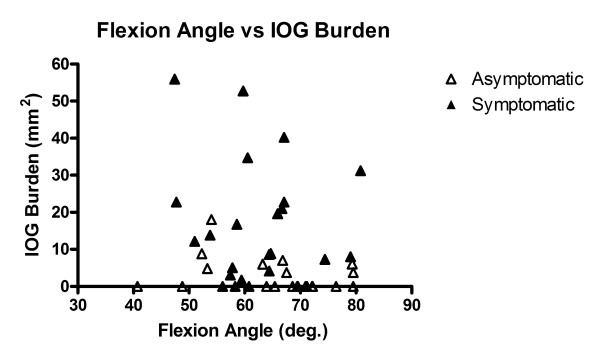
**Flexion angle versus IOG burden area**. XY plot of flexion angle versus intraosseous ganglion (IOG) burden for the asymptomatic (n = 22) and symptomatic wrists (n = 24). The correlation was not significant (p = .09).

Figure 6 shows a plot of wrist flexion angle versus total intraosseous ganglion burden area (mm^2^) for all wrists, with the exception of one asymptomatic subject with evidence of bilateral capitolunate instability and who had 14 of the 26 intraosseous ganglia identified in the asymptomatic wrists. The correlation between intraosseous ganglion burden and wrist flexion was not significant (Spearman's rho = -.250, p = .09).

## Discussion

In this study we compared the prevalence of MRI-identified abnormalities of the wrist and wrist range of motion between asymptomatic and symptomatic computer users. Similar abnormalities were frequently identified in both symptomatic and asymptomatic wrists. In only one case were the symptoms (bilateral CTS) attributable to a specific abnormality (persistent median artery within a bifurcated median nerve) [28-30]. The association between symptoms and abnormalities for the remaining wrists are less clear.

The following sub-sections describe the abnormalities that were frequently identified in symptomatic, as well as asymptomatic computer users. Repetitive biomechanical stress has been implicated in the etiology of all of these abnormalities, and there is an obvious association with computer use shown in this study.

### Extraosseous Ganglia

An extraosseous ganglion is an avascular collagen-walled sac found adjacent to, and often communicating with, joints and tendon sheaths [31]. It contains a synovial-like fluid and is similar to a synovial cyst, but is devoid of synovial cells [32,33]. The etiology of extraosseous ganglia is thought to be synovial herniation [34], mucoid degeneration of connective tissue [34], or proliferation of mucin producing cells [31].

Extraosseous ganglia were the most common abnormality; identified in 66.6% of asymptomatic and 75% of symptomatic wrists. The positive predictive value of MRI in detecting occult wrist ganglia that were confirmed histologically was 75% [22]. The prevalence of extraosseous ganglia identified with MRI has previously been reported as 19% in symptomatic wrists [35], and 51% in asymptomatic wrists [36].

There was no significant difference in the median area of extraosseous ganglia between the asymptomatic and symptomatic wrists. Extraosseous ganglia may cause symptoms due to a mass effect, but pain symptoms are often disproportionate to ganglion size [37,38]. It has been suggested that the pain associated with small extraosseous ganglia is due to pressure within the ligament [37]. Symptoms may relate to ganglion location, which were predominantly (86%) volar in a study of asymptomatic wrists [36]. In the current study, dorsal ganglia were identified in 47.8% of symptomatic wrists and in 34.8% of asymptomatic wrists, but there was nothing that differentiated between them.

According to Angelides [31] repetitive minor trauma may be an etiological factor in the development of extraosseous ganglia, but that there is no obvious correlation with occupation. However, we are not aware of any previous MRI studies of individuals employed in hand-intensive occupations, or any MRI studies of normal subjects that controlled for repetitive hand use.

Treatment of extraosseous ganglia by aspiration or excision does not alter symptom persistence when compared to no treatment for both dorsal [39] and volar [40] wrist ganglia, suggesting that symptoms originate elsewhere. In the current study, the similarities in the prevalence, size, and locations of extraosseous ganglia in asymptomatic and symptomatic wrists suggest that the symptoms are unrelated.

### Intraosseous Ganglia

Intraosseous ganglia are histologically identical to extraosseous ganglia, but are located within an osteolytic cavity [41-45]. Although similar to degenerative (osteoarthritic) cysts, intraosseous ganglia do not communicate with the joint space via eroded hyaline cartilage [46]. The etiology of intraosseous ganglia is unknown, but theories include vascular disturbance [47,48], proliferation of mucin producing cells [43], intrusion of extraosseous ganglia [47], distractive bone stress at the ligament attachments [49], or ligament degeneration [46,50].

Intraosseous ganglia were the second most common abnormality, with a greater prevalence in the symptomatic wrists (75%) compared to the asymptomatic wrists (45.8%). Not all lesions were of the characteristic spherical or oval shape, but all were located adjacent to ligament attachment sites. We cannot be certain that these lesions are true intraosseous ganglia without histological confirmation, although identification with MRI appears to be highly specific. In a cadaveric wrist study, all of the intraosseous ganglia tentatively identified with MRI were subsequently confirmed histologically [46]. The prevalence of intraosseous ganglia in that study was 9.6%, but the clinical history was unknown [46]. An MRI study of 30 asymptomatic subjects with a mean age of 31 years found 24 "bright osseous lesions" in 14 wrists, but there was no agreement on whether the lesions represented intraosseous ganglia, erosions, edema, or sub-chondral cysts [51]. A study of 400 patients with non-specific wrist pain who were screened with conventional radiography and bone scan prior to MRI found that the prevalence of intraosseous ganglia confirmed histologically was 3.7% [21].

To our knowledge there are no previous reports on the prevalence of intraosseous ganglia in a specific occupational group. The high prevalence of intraosseous ganglia in the present study may be due to the sensitivity of the inversion recovery sequence [52], as 21 of the 58 ganglia identified had a maximum dimension of less than 2 mm. The distribution of the intraosseous ganglia was very similar to previous studies, with the majority being located in the lunate, capitate, and scaphoid [46,50]. The median area of the intraosseous ganglia in the symptomatic wrists was significantly larger than in the asymptomatic wrists, but size was not a consistent determinant of symptoms.

Intraosseous ganglia have been associated with non-specific wrist pain [21], but may also be incidental findings [42]. The origin of the pain associated with intraosseous ganglia is unknown. Eiken and Jonsson [42] believe that pain results from increased intraosseous pressure only after formation of the fibrous lining, a feature also present in asymptomatic ganglia [53]. Waizenneger [54] suggests that pain is due to stretching of capsular and ligamentous tissue by a ganglion that has expanded through the cortex. However, ganglion size has not been shown to relate to symptoms [47], and 55/101 symptomatic intraosseous ganglia showed no communication with extraosseous ganglia [55]. Symptomatic intraosseous ganglia demonstrate increased radiotracer uptake [21,54,56], but this may be a non-specific finding [53,56]. Treatment of intraosseous ganglia by curettage and bone grafting is usually highly effective in relieving symptoms [21,53,57,58], although symptoms may persist without ganglion recurrence [54,59].

Intraosseous ganglia often coexist with extraosseous ganglia [50,53,59], an association we found in both asymptomatic (25%) and symptomatic wrists (58.3%). It has been suggested that chronic ligament overload is the initiating factor for the development of both intraosseous and extraosseous ganglia [50].

### Tendinopathies

Distal ECU tendon instability can result from rupture of the tendon sheath, or stripping of the sheath's ulnar attachment [60]. It is typically associated with sports such as golf and tennis, and may cause pain during forearm pronosupination [61]. Subluxation or dislocation of the distal ECU tendon was found in 58.3% of both asymptomatic and symptomatic wrists. There was no evidence of tendon rupture, and no ulnar-sided symptoms were reported by any of these subjects.

Dislocation was observed in 7 asymptomatic and 1 symptomatic wrist. However, the degree of tendon subluxation increases with forearm supination, and dislocation is normally diagnosed with the forearm fully supine [61]. In the current study forearm posture varied between neutral and supine due to imaging in the "superman" position and we cannot determine the true prevalence of dislocation.

The frequent finding of subluxation/dislocation in the current study suggests a relationship with computer use, but a longitudinal study is required. There is a possibility that ulnar-sided symptoms in this occupational group might be misattributed to distal ECU tendon instability.

Tenosynovitis is characterized as an inflammatory irritation of the tendon sheath synovium resulting in an abnormal increase in the fluid surrounding the tendon [62]. Tenosynovitis may cause pain, but may also be asymptomatic [63].

Mild tenosynovitis of the wrist extensors was identified in 2 asymptomatic and 5 symptomatic wrists.

### Wrist Range of Motion

Wrist flexion was restricted in the dominant wrist of the symptomatic group, which may be due to decreased extensibility of the wrist extensor muscles, or flexor inhibition due to pain avoidance. Only one subject reported pain at the limit of flexion, and flexion restrictions were evident in asymptomatic wrists. Restricted wrist flexion is a common finding in symptomatic computer users, which may be due to increased extensor muscle co-contraction or myofascial shortening [4,23,26]. Increased extensor muscle tension would create a substantial increase in the biomechanical loading of the muscles, tendons, and ligaments of the wrist during extremes of wrist flexion or radioulnar deviation.

There was no significant difference in radioulnar deviation between groups, which has been reported in a previous study of symptomatic repetitive workers with flexion restrictions [15]. One plausible explanation is that prolonged radioulnar deviation during computer use causes stress-induced strain of the carpal ligaments.

In contrast to our previous study [26] there was no significant decrease in wrist flexion between the prone and supine forearm postures. In that study we attributed the flexion decrease to the increase in moment arm length of the distal ECU tendon that occurs with forearm supination [64-66]. We speculate that the lack of a significant decrease observed in the current study is related to the frequent finding of ECU tendon subluxation/dislocation, which would minimize/eliminate the increase in the tendon's moment arm.

The correlation between wrist flexion and intraosseous ganglion burden did not reach significance (p = .09). However, intraosseous ganglia size is known to increase over time [42], and there may be an interaction effect. From the results of this cross-sectional study we cannot determine the length of time the flexion restrictions or the intraosseous ganglia have been present, factors that may have improved the degree of correlation.

This study is limited by the use of a convenience sample of a small number of self-referred subjects, and the lack of histological confirmation of the intraosseous ganglia. The severity of symptoms varied, and the results may not generalize to computer users with severe, disabling symptoms. Inclusion of two subjects with previous wrist surgery is a possible confound for our results.

## Conclusions

This appears to be the first MRI study of wrist abnormalities in computer users.

This study demonstrates that a variety of wrist abnormalities are common in computer users and that only intraosseous ganglia prevalence and size differed between asymptomatic and symptomatic wrists. Flexion was restricted in the dominant wrist of the symptomatic group, but the correlation between wrist flexion and intraosseous ganglion burden did not reach significance. Wrist flexion restriction may be an indicator of increased joint loading, and identifying the cause may help to guide preventive and therapeutic interventions.

## Competing interests

The authors declare that they have no competing interests.

## Authors' contributions

RB and RT designed the study, RB performed data collection and statistical analysis, and WP read the MR images. All authors were involved in drafting and revising the manuscript, and have read and approved the final manuscript.

## Pre-publication history

The pre-publication history for this paper can be accessed here:

http://www.biomedcentral.com/1471-2474/11/273/prepub
